# Dentists' attitudes and prescription practices for analgesics and antibiotics in Kirkuk Governorate, Iraq

**DOI:** 10.25122/jml-2023-0405

**Published:** 2023-12

**Authors:** Zainab Azal Mahdi, Jihan Abdulhussein Ibrahim

**Affiliations:** 1 Department of Basic Sciences, College of Dentistry, University of Kirkuk, Kirkuk, Iraq; 2 Department of Oral Surgery, College of Dentistry, University of Kirkuk, Kirkuk, Iraq

**Keywords:** antibiotics, empirical therapy, analgesics, non-steroidal anti-inflammatory drugs, mefenamic acid, paracetamol, diclofenac, ibuprofen

## Abstract

The inappropriate use of analgesics and antibiotics is a widespread issue among dentists globally, leading to the risk of over-prescription that could negatively affect patient health and quality of life. This study aimed to assess the prescribing patterns of analgesics and antibiotics by dentists in Kirkuk City, Iraq, focusing on their attitudes, knowledge levels, and practices regarding these medications. A cross-sectional survey was conducted among 280 dentists in Kirkuk City. The dentists were contacted via their work email addresses, and they responded to a survey. Descriptive statistics, including frequency analysis, were employed to evaluate the appropriateness of analgesic and antibiotic prescriptions for different dental conditions. The first-choice analgesic for 44.6% of dentists was mefenamic acid, followed by paracetamol (31.1%). Regarding antibiotic use, 56.8% of dentists in Kirkuk City reported using antibiotics for empirical and direct therapy. Other dentists (43.2%) revealed that they did not have enough information regarding antibiotic group preference in empirical therapy. 106 of the participants (37.85%) recommended the use of broad-spectrum antibiotics in the treatment of bacterial infections. However, most (45%) were unfamiliar with the group preferences in empirical therapy. Dentists in Kirkuk City showed variations in knowledge and awareness regarding using analgesics and antibiotics. This requires further education and training on proper analgesics and antibiotic stewardship guidelines.

## INTRODUCTION

Dental pain associated with dental or periodontal disease is a common reason for patients to seek dental care. Additionally, many dental procedures can be painful, and postoperative pain may persist for several days. Therefore, dentists should prescribe suitable analgesics to alleviate dental pain and manage discomfort associated with inflammation or surgery. The management of pain typically follows the 3 "D" principle, which includes diagnosis, dental treatment, and drugs [1].

Various classes of analgesics are available, including opioids and non-opioid analgesics. Opioids, while effective, are associated with serious adverse effects such as respiratory depression, sleep disturbances, sedation, dependence, and addiction. Therefore, their use is generally limited to managing severe cases [2].

Non-steroidal anti-inflammatory drugs (NSAIDs) are effective and commonly used to address initial pain of inflammatory origin, providing analgesia for mild to moderate pain. Dentists frequently prescribe this class of analgesic to improve clinical outcomes. NSAIDs work by suppressing inflammatory pain by inhibiting cyclooxygenase (COX) enzymes, which play a primary role in the generation of prostaglandins, mainly prostaglandin E2 (PGE2), that contribute to inflammatory pain [3]. NSAIDs are a group of chemically dissimilar agents that differ in their potency and effects on renal, cardiovascular, and gastrointestinal tract systems. Dentists should know these differences to make informed choices regarding the safest and most appropriate drug for a clinical condition [4].

In routine dental practice, bacterial infections frequently manifest as symptoms of swelling and pain in the oral cavity. Treatment of these orofacial infections is mandatory as they may lead to serious and irrecoverable consequences such as dentoalveolar abscess, airway obstruction, brain abscess, sinusitis, orbital abscess, and blindness [5]. Strategies for managing dental infection involve surgical interventions, endodontics therapy, and prescription of antibiotics. Current guidelines emphasize the importance of first eliminating the source of infection through surgical procedures, followed by a short course of antibiotics lasting 2-3 consecutive days. Prolonged antibiotic therapy is not recommended, as it has not been shown to provide significant benefits and may lead to adverse outcomes [6].

Prior studies in Iraq have indicated that the frequency of correctly prescribed antibiotics for necessary cases and the misprescription of antibiotics for unnecessary orofacial conditions are nearly the same. Unnecessary antibiotic prescriptions could result in severe complaints such as oral bacterial resistance, which represents a growing concern in dentistry and medicine. Additionally, antibiotic use has been associated with gastric and hematological problems and alterations in bacterial microbiota. Antibiotics should be prescribed in accordance with guidelines to prevent these issues [7, 8]. This study aimed to examine the attitudes and knowledge of dentists regarding prescription patterns of analgesics and antibiotics in dental practice. To our knowledge, this is the first study conducted in Kirkuk City, Iraq, to examine this aspect.

## MATERIAL AND METHODS

### Study design and setting

This cross-sectional study included dentists from various sectors in Kirkuk City, Iraq. A digital version of the questionnaire was created using Google Survey and distributed to a random selection of dentists via their work email addresses. The email addresses were obtained from the database at Kirkuk Dental Council, and respondents were requested to submit their responses by a specified deadline. In total, 280 valid responses were received and subsequently analyzed. The research was conducted under the supervision of the College of Dentistry at the University of Kirkuk. The questionnaire was distributed between October 1, 2022, and January 1, 2023.

### Questionnaire

The questionnaire was adapted from previous studies [7, 9, 10] with necessary modifications to align with the specific objectives of this investigation. It was developed and administered through Google Survey and consisted of three main sections:

1. The first section included information regarding dentists’ age, gender, educational level, years of experience, and area of employment.
2. The second section was designed to gather data about analgesic-prescribing patterns using criteria as an indication for the use of analgesics in dentistry, most prescribed analgesics, factors considered during drug prescription, and dentist awareness of drug interaction.
3. The third section focused on the level of dentist knowledge about antibiotics, the basis of antibiotic prescription, the preferred antibiotic group based on the spectrum of activity in empirical therapy, and whether susceptibility tests were conducted when prescribing antibiotics.

### Inclusion and exclusion criteria

Dentists of both genders registered in the Iraqi Dental Association as a part of the Kirkuk Dental Council and currently working in the public and/or the private sector inside Kirkuk City were included in the study. Dentists who did not consent or submitted their forms after the due date were excluded from the study.

### Sample size estimation and randomization

The total number of practicing dentists in Kirkuk Governorate, estimated at 433 by the Kirkuk Dental Council, constituted the population (n). The sample size was determined with a 95% confidence level, 5% margin of error, and 50% response distribution.


$$\begin{array}{cc} Sample\;size & =\frac{Distribution\;of50\%\;}{{\left(\frac{M\arg in\;of\;error\%}{Confidence\;level\;score}\right)}^{2}}\\ True\;sample & =\frac{Sample\;size\;X\;Population}{Sample\;size+Population-1} \end{array}$$

A random selection of 350 dentists was made from the official email list of the Kirkuk Dental Council using Research Randomizer. The final sample size was adjusted to 280 to account for potential non-responses.

### Statistical analysis

Data collected from the questionnaire were recorded and analyzed using the Statistical Package for Social Sciences^®^ (SPSS), version 25.0. The response rate for each question was calculated as a percentage of total responses. Frequency analysis was used to examine demographics and patterns of analgesic and antibiotic prescription.

## RESULTS

### Participant demographics, academic background, and professional experience

The online questionnaire comprised demographic information such as area of employment, years of experience, qualifications, age, and gender (Table 1). A total of 280 dentists (51.8% female and 48.2% male) responded to the questionnaire, giving a response rate of 100%. Most participants were general practitioners (91.4%) with 1-5 years of practice (68.9%) working at the health directorate (55.35%).

**Table 1:** Socio-demographic characteristics of the study population

Variable	n (%)	
**Gender**		
Male	135 (48.2%)	
Female	145 (51.8%)	
**Age (years)**		
20–30 years	212 (75.7%)	
31-40 years	58 (20.7%)	
41<years		10 (3.6%)
**Years of practice (years)**		
1-5 years	193 (68.9%)	
6-10 years	63 (22.5%)	
>10 years	24 (8.6%)	
**Qualification**		
General practitioner	256 (91.4%)	
Higher degree	24 (8.6%)	
**Area of employment**		
University	77 (27.5%)	
Health directorate	155 (55.35%)	
Private clinics	48 (17.15%)	

### Prescription pattern of analgesics

**Figure 1:**
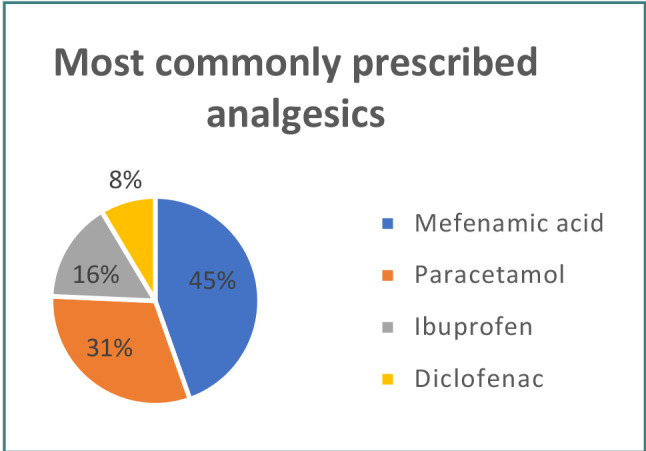
Most prescribed analgesics by dentists in Kirkuk City

Table 2 demonstrates the prescription pattern of analgesics by dentists in Kirkuk City. The majority of respondents (51.8%) prescribed analgesics for managing dental pain, while 22.5% prescribed them to prevent postsurgical complications, 20.7% for inflammation and diffuse swelling, and 5% for fever. Mefenamic acid was the most commonly prescribed analgesic (44.6%), followed by paracetamol (31.1%), ibuprofen (15.7%), and diclofenac (8.6%). Figure 1 showcases the most described analgesics by dentists in Kirkuk City.

Regarding prescription considerations, 72.5% of participants took into account a patient's history of cardiovascular, renal, and gastrointestinal disorders as a crucial factor when prescribing analgesics. Some respondents focused on specific conditions, such as peptic ulcers (15.3%), hypertension (8.6%), or renal disease (3.6%), when considering analgesic prescriptions. Most respondents (75.7%) regarded being aware of potential drug interactions as a crucial aspect of analgesic prescription to prevent complications associated with this class of medications.

**Table 2:** Prescribing patterns of analgesics among Iraqi dentists in Kirkuk City

Variable	n (%)
**The main indication for analgesics in dentistry**	
Dental pain management	145 (51.8%)
Prevention of postsurgical complication	63 (22.5%)
Diffuse swelling	58 (20.7%)
Fever	14 (5%)
**Most commonly prescribed analgesics**	
Mefenamic acid	125 (44.6%)
Paracetamol	87 (31.1%)
Ibuprofen	44 (15.7%)
Diclofenac	24 (8.6%)
**Patient factors to be considered during analgesic prescription**	
History of hypertension	24 (8.6%)
History of peptic ulcer	43 (15.3%)
History of renal diseases	10 (3.6%)
All the factors mentioned above (hypertension, peptic ulcer, and renal diseases)	203 (72.5%)
**Awareness about drug interaction is crucial in the drug prescription act**	
Strongly agree	106 (37.85%)
Agree	106 (37.85%)
Neutral	63 (22.50%)
Disagree	5 (1.8%)
Strongly disagree	0 (0.0%)

### Prescription pattern of antibiotic

Most respondents (79.3%) indicated that their knowledge of antibiotics generally ranged from fair to good (Table 3). Regarding antibiotic dosage and duration, 73.2% of respondents adhered to standard guidelines, while 18.2% prescribed antibiotics on a case-by-case basis. Most dentists (56.8%) responded that they would use antibiotics in both empirical and direct therapy following the specification of infection-causing bacteria. However, a small number (3.6%) responded that the base of antibiotic use is for empirical therapy without the need for bacterial species identification.

**Table 3:** Prescribing patterns of antibiotics among Iraqi dentists in Kirkuk City

Variable	n (%)
**Self-awareness of antibiotics knowledge among dentists**	
Poor	53 (18.9%)
Fair	92 (32.9%)
Good	130 (46.4%)
Very good	0 (0.0%)
Excellent	5 (1.8%)
**Do you follow standard guidelines for duration and dosage when prescribing antibiotics**	
Yes	205 (73.2%)
No	24 (8.6%)
Differs with each case	51 (18.2%)
**Basis of antibiotic prescription**	
Direct therapy	68 (24.3%)
Empirical therapy	10 (3.6%)
Combination of both direct and empirical therapy	159 (56.8%)
Insufficient knowledge	43 (15.3%)
**Preferred antibiotic group based on the spectrum of activity for empirical therapy**	
Broad spectrum	106 (37.85%)
Extended-spectrum	19 (6.8%)
Narrow spectrum	29 (10.35%)
Insufficient knowledge	126 (45%)
**Frequent conditions for antibiotic prescription**	
Odontogenic infection	60 (21.4%)
Periodontal infection	72 (25.7%)
Prophylactic use	121 (43.2%)
Patient satisfaction	27 (9.7%)
**Importance of performing a susceptibility test when prescribing antibiotics**	
Strongly agree	91 (32.5%)
Agree	116 (41.4%)
Neutral	63 (22.5%)
Disagree	5 (1.8%)
Strongly disagree	5 (1.8%)
**Necessity of testing for hypersensitivity reaction before antibiotics prescription**	
Yes	106 (37.9%)
No	97 (34.6%)
Maybe	41 (14.6%)
Insufficient knowledge	36 (12.9%)

Regarding empirical therapy, 126 of the respondents (45%) were not sure and did not have enough knowledge about the preferred antibiotic group in terms of the spectrum of activity. In contrast, 106 dentists preferred the prescription of broad-spectrum antibiotics, and 48 (17.1%) preferred extended and narrow spectrum for empirical treatment of orofacial bacterial infections.

In terms of common indications for antibiotics, 43.2% of the dentists mentioned that they often prescribe antibiotics prophylactically based on the procedure to be performed. In addition, 25.7% responded that they would prescribe this medication for periodontal infection, 21.4% of dentists would prescribe it to manage odontogenic infection, and 9.6% of dentists responded that patient satisfaction constituted a ground for antibiotic prescription.

A significant proportion of participants (73.9%) emphasized the importance of performing culture sensitivity and susceptibility testing before initiating direct therapy or during the commencement of empirical therapy to ensure appropriate antibiotic prescription. Only five dentists disagreed with this approach. An interesting finding was that 37.9% of the dentists tested the patient for hypersensitivity before prescribing antibiotics, and 34.6% neglected the need for such tests. A minority of respondents (12.9%) did not know enough about such test performance.

## DISCUSSION

To our knowledge, this was the first survey to assess analgesics and antibiotics prescription patterns among dentists in Kirkuk City. The current literature on this subject within research databases is quite limited. A relevant study conducted by Zaid *et al*. in 2022 addressed the pattern of systemic antibiotic prescriptions by Iraqi dentists but did not specifically isolate the practices within Kirkuk City [7].

The prescription of analgesics and antibiotics has been a significant component of dental care in recent years [11]. Intraoral inflammation can lead to swelling and pain, the most cited oral health−related reasons for patients to consult a dentist worldwide [12]. However, a significant challenge in prescribing these medications among dentists is insufficient knowledge and awareness. Many dentists do not invest as much effort in understanding and implementing the latest clinical guidelines for these medications as they do in hands-on training and skill-based learning. This may be attributed to a lack of awareness about the importance of evidence-based guidelines and a shortage of training in this specific aspect [13].

In this research, we conducted a survey among dentists in Kirkuk and analyzed their responses to gain insights into how they prescribe analgesics and antibiotics. Most dentists (51.8%) said they prescribe analgesics to address dental pain from different causes since most patients experience acute or chronic pain. Other respondents preferred the use of analgesics as prophylaxis for post-surgical complications and diffuse swelling as they proved to be effective when prescribed at total therapeutic doses, being even superior to single-entity immediate-release opioid formulations of morphine codeine and oxycodone in the management of pain after the surgical removal of impacted third molar teeth [14, 15]. As NSAIDs, analgesics have anti-inflammatory effects along with pain reduction properties that can be used as prophylaxis to ameliorate expected post-surgical complications like pain and swelling.

Dentists in Kirkuk City commonly prescribed mefenamic acid (44.6%), followed by paracetamol (31.1%), ibuprofen (15.71%), and diclofenac (8.6%). The utilization of these analgesics aligns with findings from several studies, which confirm their efficacy in providing excellent pain relief for moderate to severe discomfort, as well as their anti-inflammatory effects [16, 17].

Despite its lower anti-inflammatory effects on peripheral tissues compared to other NSAID analgesics, dentists frequently prescribe paracetamol. This study confirms its popularity among dentists mainly because it has several advantages, such as a lower risk of gastrointestinal bleeding, safety during pregnancy, and fewer interactions with other medications [16].

Patient factors are very important and must be considered during the prescription of NSAIDs as this class of analgesics is known to impose risks to the gastrointestinal tract, cardiovascular, and renal system and is associated with 30% admission to the hospital for avoidable adverse drug reactions and adverse effects [18]. This study revealed that a large number of respondents (75.7%) were aware of the multiple cytotoxic effects of NSAIDs analgesics while prescribing this class of analgesic, 15.3% of respondents considered the cautious prescription of NSAIDs analgesics in patients with only a history of peptic ulcer and GIT bleeding neglecting other systemic side effects.

A significant proportion of patients who take NSAIDs, whether prescribed by a physician or obtained over-the-counter, may also be receiving treatment for other health conditions, raising the potential for adverse drug reactions. Although serious drug interactions with NSAIDs are rarely reported, this is likely attributable to the typically short-term use of these medications. However, it remains important not to overlook the potential for such interactions [19]. Among the survey respondents, 75.7% recognized the importance of considering possible drug interactions when prescribing NSAIDs, while (22.5%) of the participants had a neutral opinion, possibly under the assumption that the risk of interaction is minimal in young and healthy individuals.

Orofacial infections can be categorized as either odontogenic, originating from tooth tissue, or non-odontogenic. Most orofacial infections in humans are of odontogenic origin [20]. Therefore, antibiotics prescribed by dentists for the treatment or prevention of infections have become one of the important aspects of dental practice and account for the vast majority of medications prescribed by dental practitioners [21]. More than 700 species of bacterial flora are naturally occurring in the oral cavity. The most prevalent bacterial genus is gram-positive cocci Streptococcus, encompassing species such as Streptococcus mitis, Streptococcus sanguinis, Streptococcus salivarius, and Streptococcus anginosus. These species are known contributors to developing odontogenic infections [22]. It was noted that gram-positive cocci are the causative agents for about 65% of orofacial infections since they are the most common microflora in the oral cavity, and gram-negative bacilli could be found in 25% of oral specimens collected [23].

The main indications for antibiotic prescription in dentistry are the treatment of odontogenic infections, treatment of nonodontogenic infections, prevention of focal infection, and prevention of local infections, as well as other specific conditions like immunocompromised patients [24]. According to previous studies on bacterial susceptibility to antibiotics in odontogenic infections, almost 70% of bacteria isolated were susceptible to penicillin [25]. Many factors have rendered penicillin the first-line drug of choice for treating odontogenic infections, such as its cost-effectiveness, appropriate antimicrobial activity, and low incidence of adverse effects [26, 27]. If a patient has a history of hypersensitivity to penicillin, clindamycin is recommended [28].

The most frequently prescribed antibiotics in dentistry, listed in descending order of prescription rate, include amoxicillin, amoxicillin + clavulanic acid, clindamycin, azithromycin, and clarithromycin [29]. Amoxicillin is the most commonly prescribed antibiotic among Iraqi dentists, followed by amoxicillin + clavulanic acid (co-amoxiclav) [7]. Amoxicillin is one of the penicillin antibiotic families that acts mainly against gram-negative bacteria [30, 31]. The dosage for amoxicillin is 500 mg every 8 hours or 1 g every 12 hours [26]. Amoxicillin with clavulanic acid (co-amoxiclav) is considered a broad-spectrum antibiotic. In cases of severe infections, a high dose of co-amoxiclav (875/125 mg every 8 hours or 2000/125 mg every 12 hours) is recommended [32]. Dentists should be aware of the hepatotoxicity adverse effects that could result from this medication [25].

Antibiotic prescription is associated with unfavorable adverse effects, including hypersensitivity, dermatological reactions, gastrointestinal disturbances, hematological complications, and alterations in bacterial microbiota. In recent years, bacterial resistance has been increasingly linked to the overuse or misuse of broad-spectrum antibiotics [8, 22, 33].

The British Society for Antimicrobial Chemotherapy has indicated that the misuse or inappropriate use of antibiotics contributes to the rise of antibiotic-resistant strains compared to their susceptible population, leading to increased resistance. This increase in resistant strains invariably contributes to an enormous increase in infection-related mortality rates [34]. Recently, we have witnessed a concerning trend where certain bacterial species have developed resistance to all existing antibiotics [21]. The prevalence of resistance development is increasing in Kirkuk City [35, 36], which has encouraged the assessment of antibiotic prescription in dental practice in this study.

To prevent the emergence of antibiotic-resistant strains and their associated risks, studies recommend prescribing antibiotics with a narrow spectrum and only after identifying the causative bacteria [24]. In this study, most dental practitioners (81.1%) indicated that they have acquired basic knowledge and understanding about antibiotics to deal with such conditions confidently, and most of them (73.2%) follow standard guidelines for antibiotic dosage and duration. Contrastingly, previous research has demonstrated that only around 12% of dentists correctly and adequately prescribe antibiotics for treatment or prophylaxis [37]. More than half of the dentists who answered the questionnaire (56.8%) preferred using antibiotics in empirical and direct therapy of bacterial infections following the specification of the bacteria. However, a minority (3.6%) support using antibiotics for empirical therapy based only on clinical experience, without confirmation of the bacterial strain. These responses suggest an awareness of both empirical and direct antibiotic therapy among dentists, though empirical prescribing practices may overlook potential adverse effects resulting from the obsolete use of these medications [34].

Many respondents (45%) lack adequate knowledge about selecting the appropriate antibiotic group based on the activity spectrum for empirical treatment of bacterial infections. This points to a need for better-structured strategies and increased educational efforts in dental antibiotic prescribing to enhance familiarity with these drugs and improve prescribing patterns [38]. In this study, 38% of participants chose the prescription of broad-spectrum antibiotics, and a small number (17.1%) preferred extended and narrow spectrum for empirical therapy of oral bacterial infections.

Although not all odontogenic infections need antibiotic therapy, it is crucial not to substitute antibiotics for essential dental procedures such as incision and drainage, debridement, or endodontic treatment. When systemic antibiotic therapy is necessary, it should follow these initial treatments [32, 39].

Many dentists (43.2%) prescribe antibiotics for prophylaxis, which may not be necessary. This aligns with findings from previous research indicating that over 80% of such prescriptions in dental settings were unnecessary in dental practice [40]. An explanation for this practice could be an intention to prevent infection during endodontic procedures. However, the prophylactic use of antibiotics is not well supported and gave rise to inconsistent results [41]. A small percentage of respondents (9.5%) would prescribe antibiotics for nonclinical factors such as patient demand [21]. The study results are inconsistent with many studies that reported unnecessary and excess use of antibiotics in conditions where antibiotics may not be required [42, 43].

Among the surveyed dentists, 207 agreed on the importance of conducting bacterial susceptibility testing to guide the appropriate prescription of antibiotics, whether for direct or empirical therapy. Only 5 dentists questioned the necessity of these tests, possibly relying on their familiarity with common oral bacteria and their own clinical experience.

Participants' responses regarding the performance of hypersensitivity reaction tests, considered an important tool of antibiotic stewardship, varied [44]. Some preferred interpreting such a test (37.9%), while for others (34.6%) it was unnecessary.

The current study has certain limitations that need to be considered. Its cross-sectional design and the use of an internet-based questionnaire may introduce potential biases. Furthermore, the study lacked detailed information on the specific types of antibiotics used and the guidelines for prescribing antibiotics in dental practice in Kirkuk.

## CONCLUSION

The study highlights the variability in dentists' knowledge and understanding of analgesics and antibiotic prescriptions in Kirkuk City. This variability may be attributed to a lack of scientific awareness of medication pharmacology and the challenge of translating evidence-based information into clinical practice. To address this issue, there is a need for prescription training based on clear and evidence-based guidelines to be incorporated into the clinical training of dentists. It is essential to increase awareness among dentists about the potential consequences of incorrect prescriptions, especially since dental clinic visits often involve pain or inflammation-related issues.
